# Impacts of Climate Change and Human Activity on the Potential Distribution of *Conogethes punctiferalis* in China

**DOI:** 10.3390/insects16100998

**Published:** 2025-09-25

**Authors:** Cheng-Fei Song, Qing-Zhao Liu, Jiao Liu, Xin-Yao Ma, Fa-Lin He

**Affiliations:** 1 Shanxi Center for Testing of Functional Agro-Products, Shanxi Agricultural University, Taiyuan 030031, China; 2 Department of Entomology, Nanjing Agricultural University, Nanjing 210095, China; qzliu98@163.com

**Keywords:** *Conogethes punctiferalis*, MaxEnt, human activity, climate change, potential distribution

## Abstract

*Conogethes punctiferalis* (Guenée, 1854) is a polyphagous pest with a wide host range and strong reproductive ability, and its potential threat to agricultural production cannot be ignored. Based on the optimized maximum entropy niche model, this study evaluated potential suitable habitats for *C. punctiferalis* in China and their dynamic changes under current conditions (Model 1: bioclimatic factors + elevation; Model 2: bioclimatic factors + elevation + human activity) and four different future climate scenarios (Model 3: bioclimatic factors + elevation + human activity). The results suggest that the potential suitable habitats for *C. punctiferalis* are mainly driven by a combination of temperature, precipitation, elevation, and human activity. Under current conditions, suitable habitats are mainly concentrated in southern Northeast China, North China, the Yangtze River Basin, and its south regions; highly suitable areas are primarily located in the main maize-producing regions of the Huang-Huai-Hai Plain. The area of suitable habitats predicted by Model 2 is smaller than that predicted by Model 1. Under future climate scenarios, the potential distribution range of *C. punctiferalis* will show an expanding trend, with the expanded area larger than the contracted area. Compared with Model 2, the suitable areas are expected to increase under Model 3 by approximately 91,799 km^2^ to 723,711 km^2^. This study provides an important basis for assessing the potential hazard risk of *C. punctiferalis* and is of major significance in guiding the formulation of targeted integrated pest management strategies and protecting the safety of agricultural production.

## Simple Summary

The polyphagous pest *Conogethes punctiferalis* poses a significant threat to agricultural production. This study used the optimized maximum entropy model, combined with bioclimatic variables, elevation, and human activity, to predict potential suitable areas for *C. punctiferalis* in China, as well as its spread patterns. The results indicate that suitable habitats are mainly concentrated in southern Northeast China, North China, the Yangtze River Basin, and its south regions. Including anthropogenic factors reduced the predicted suitable areas compared to using only bioclimatic data and elevation. The potential geographical distribution of this pest is gradually expanding in China. This research provides references for early warning and management in the control of *C. punctiferalis*.

## 1. Introduction

*Conogethes punctiferalis* (Guenée, 1854) (Lepidoptera: Pyralidae), commonly known as the yellow peach moth, is a major agricultural pest with wide distribution across Southeast Asia and Australia and established populations in Europe and the United Kingdom [1]. This polyphagous pest has been documented as damaging over 100 different host plants, including fruits, vegetables, grains, and ornamental plants [2,3,4]. In recent years, the population of *C. punctiferalis* has been increasing continuously, and its widespread harmfulness and destructiveness pose a severe threat to the fruit industry and field crops in temperate to tropical regions worldwide. The pest primarily inflicts substantial damage on host plants through larval boring and feeding [5]. In fruit trees, larvae bore into the fruits, impeding their normal development and causing discoloration and abscission. The infested fruits develop meandering internal tunnels that are filled with frass. In mild infestations, this results in a drastic decline in fruit quality and the loss of commercial value; in severe cases, it triggers fruit drop and rot, leading to complete crop failure. In agricultural crops, such as maize, *C. punctiferalis* has emerged as one of the major pests in China, with its damage severity surpassing even that of *Ostrinia furnacalis* (Lepidoptera: Crambidae) [2,6]. Its larvae bore into maize ears to feed on kernels, inducing severe ear rot, and also bore into stems, causing plant lodging. These damages collectively lead to direct economic losses [7,8,9]. *C. punctiferalis* has adverse impacts on China’s forestry and fruit industry and agriculture, posing a severe threat to the economic success and sustainable development of related sectors. With the changes in the agricultural industrial structure and the expansion of planting areas for high-value-added cash crops, the potential scope for damage and economic impacts of this species are further escalating. Moreover, this pest has strong cross-border dispersal potential and ecological adaptability, which have rendered it a quarantine pest of significant concern in global agricultural product trade [10].

The Global Mean Surface Temperature (GMST) is projected to increase by at least 1.5 °C during the period 2021–2040, with a rate of temperature increase that is more than twice the observed rate over the past 100 years [11]. Global climate change has already exerted extensive impacts on ecosystems, causing alterations in species’ distribution ranges and occurrence patterns [12,13]. It is projected that such impacts will intensify over the next 20 years [14]. Studies have indicated that climate warming can induce changes in species distribution through alterations in population growth rates, propagule pressures, and dispersal patterns [15,16]. Therefore, a comprehensive understanding of species’ responses to climate change is crucial in both ecological research and practical applications [17]. By predicting the potential distribution range of pests, preventive measures can be implemented in advance to reduce their occurrence, mitigate economic losses, and protect the agricultural and ecological environment.

Species distribution models (SDMs) have been widely applied in scientific environmental research, biodiversity conservation, and natural resource management [18,19]. Currently, models used to predict the potential suitable distribution of species include MaxEnt (maximum entropy), CLIMEX/DYMEX, DOMAIN, GARP, BIOCLIM, and DIVA-GIS. These models predict species’ suitable habitat ranges under current and future climate conditions by computationally defining their environmental requirements based on occurrence and environment data and scaling these requirements across time and space [20]. Among various predictive models, the MaxEnt model has been widely applied in ecology, conservation biology, evolutionary biology, biogeography, and climate change research due to advantages such as insensitivity to sample size, simplicity of operation, and high prediction accuracy [21]. It is used to assess the potential impacts of climate change on species [22] and also to study the conservation of rare and endangered species, as well as the potential expansion areas of invasive alien plants [23,24].

Climatic factors, such as temperature, precipitation, and humidity, are important determinants of pest distribution. However, with the intensification of human activities, factors including land use change, agricultural activities, and urbanization have emerged as critical and non-negligible drivers of pest dispersal and colonization. Agricultural practices such as irrigation, fertilization, and pesticide application can alter the living environment of pests, directly or indirectly affecting species’ spatial distribution and diversity [25,26]. Furthermore, *C. punctiferalis* has a broad range of host plants, and human activities related to the cultivation, transportation, and trade of these hosts have created favorable conditions for its long-distance dispersal, enhancing its potential for cross-regional spread [27,28]. For instance, the transregional transportation of fruits and crops may introduce *C. punctiferalis*-infested fruits or plants into new areas. To address these challenges, studying the impacts of both climate change and human activities on species’ geographical distribution patterns can more accurately and realistically reflect the actual distribution of *C. punctiferalis*. This will facilitate a more precise assessment of its potential distribution range and represent a scientific basis for pest management.

For the economically significant *C. punctiferalis*, previous studies have mainly focused on biological characteristics [29,30], comprehensive control [31,32], genomics [33,34], and pheromone [35,36]. As of yet, however, no research has been undertaken to forecast the potential habitat distribution of *C. punctiferalis* via the SDM, and the critical factors influencing the distribution patterns of this pest are yet to be elucidated. Based on this, the present study is based on existing geographical distribution data for *C. punctiferalis*. By means of variable screening and with an optimized MaxEnt model, this research systematically explores the effects of climate change and human activities on its habitat distribution. The specific objectives are as follows: (1) to compare the differences in the habitat distribution of *C. punctiferalis* between scenarios with and without human disturbances; (2) to explore the changes in the habitat distribution patterns of *C. punctiferalis* under different climate scenarios; (3) to shed light on spatial variation and development trends in *C. punctiferalis*. These findings will help further delineate its distribution traits and shifting patterns across China, provide theoretical support for the formulation of scientific and feasible pest management strategies to cope with the challenges brought about by global climate change, reduce the harm caused by *C. punctiferalis* in agricultural production, and ensure food security and the sustainable development of the ecological environment.

## 2. Materials and Methods

### 2.1. Occurrence Points of C. punctiferalis

**Figure 1:**
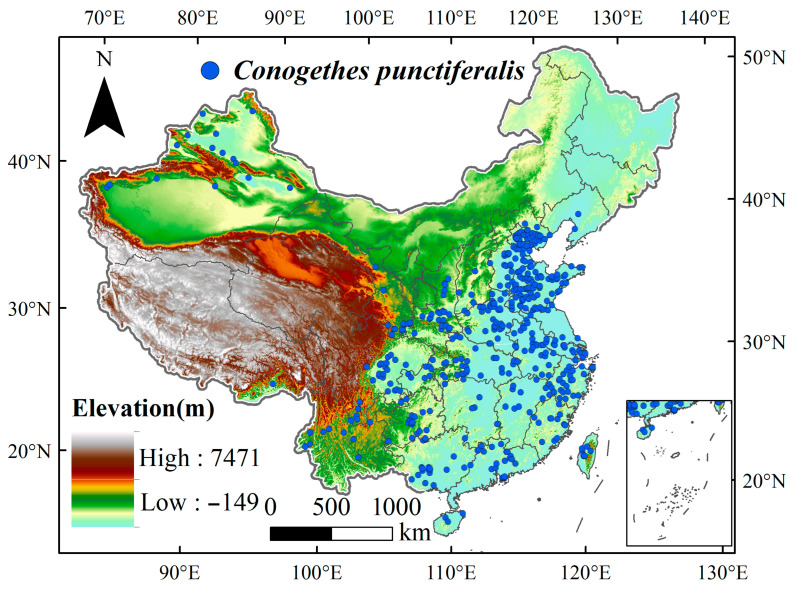
Occurrence records for the *C. punctiferalis* population in China.

The occurrence locations of *C. punctiferalis* were sourced from the published literature and online databases, including Web of Science (accessed on 26 June 2025, at https://www.webofscience.com/wos/), EPPO (accessed on 27 June 2025, at https://gd.eppo.int), CABI (accessed on 27 June 2025, at https://plantwiseplusknowledgebank.org/), CNKI (accessed on 22 June 2025, at https://www.cnki.net/), and GBIF (accessed on 27 June 2025, at https://www.gbif.org/). Data points exhibiting duplicate latitude/longitude pairs or unidentifiable coordinates were removed. For distribution points lacking specific geographic coordinates, the corresponding latitude and longitude information was obtained using Google Earth (accessed on 28 June 2025, https://earth.google.com/web/). To prevent sampling bias and record redundancy from impacting the accuracy of model predictions, species distribution records were processed via ENMTools 1.4, with the aim of retaining only one distribution point within each grid at a spatial resolution of 2.5 arc-min (approximately 4.5 km^2^). The final dataset included 466 distribution points, which were used for model training and validation (Figure 1).

### 2.2. Environmental Variables

The model integrates multiple environmental factors such as climate variables, elevation, and anthropogenic data to explain the geographical distribution of *C. punctiferalis* in China. Climate data encompassing 19 bioclimatic variables at 2.5 arc-min resolution were acquired from WorldClim v2.1 for three periods: 1970–2000 (near current), 2041–2060 (2050s), and 2061–2080 (2070s). Future climate data for the 2050s and 2070s were obtained from the BCC-CSM2-MR General Circulation Model (GCM), based on four Shared Socioeconomic Pathways (SSP1-2.6, SSP2-4.5, SSP3-7.0, and SSP5-8.5). This model has the ability to simulate the long-term evolution of temperature and precipitation data across China [37]. Elevation data (2.5 arc-min resolution) were acquired from WorldClim (https://worldclim.org/download from 10 April 2025). Anthropogenic variables, including the Human Influence Index (HII) at 1 km^2^, were sourced from the Global Human Influence Index v2 (1995–2004) [38]. Two time periods (2050s and 2070s) were combined with four SSP scenarios to generate eight future climate scenarios: SSP1-2.6-50, SSP1-2.6-70, SSP2-4.5-50, SSP2-4.5-70, SSP3-7.0-50, SSP3-7.0-70, SSP5-8.5-50, and SSP5-8.5-70.

**Figure 2:**
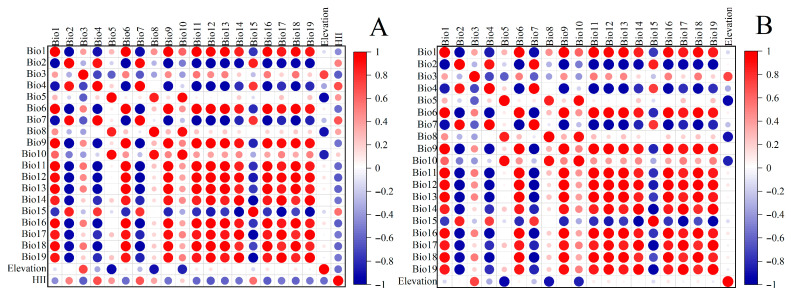
Pearson correlation coefficient analysis between *C. punctiferalis* and environmental variables with (**A**) and without (**B**) interference from human activities.

In addition, we assessed variable importance using the Jackknife method and quantified pairwise collinearity among environmental predictors through Pearson correlation analysis (SPSS software 24.0). This identified statistically significant correlations across the 19 bioclimatic variables, elevation, and HII. When the correlation coefficient of 2 variables was |*r*| > 0.8, we only retained the variables with a higher percentage contribution (Supplementary Table S1) [39]. The final predictor selection prioritized variables demonstrating both a high model contribution (Jackknife) and low multicollinearity (|*r*|≤ 0.8), ensuring ecological interpretability in distribution modeling [40]. Ultimately, Model 1 was built using 7 environmental variables: the mean diurnal range (bio2), isothermality (bio3), minimum temperature of the coldest month (bio6), mean temperature of the wettest quarter (bio8), precipitation in the driest month (bio14), precipitation seasonality (bio15), and elevation (Figure 2B). Model 2 was built using 7 environmental variables: the mean diurnal range (bio2), isothermality (bio3), minimum temperature of the coldest month (bio6), precipitation in the driest month (bio14), precipitation seasonality (bio15), elevation, and HII (Figure 2A).

### 2.3. Model Analysis Optimization

All chosen variables were resampled to a consistent spatial resolution of 2.5 arc-min via the bilinear interpolation method. To examine the effects of environmental changes and human influence on the distribution patterns of *C. punctiferalis*, three models were constructed, as follows: Model 1 predicts species distribution using 20 environmental variables (19 bioclimatic factors + elevation) under current climate conditions; Model 2 predicts species distribution using 21 environmental variables (19 bioclimatic factors+ elevation + HII) under current climate conditions; and Model 3 predicts species distribution using 21 environmental variables (19 bioclimatic factors + elevation + HII) under future climate conditions. Models 1 and 2 both utilize current climate projections, while Model 3 employs future climate projections. The comparison between Model 1 and Model 2 reflects the impact of human activities on species’ suitable habitats. Comparing Model 2 and Model 3 can reveal the impact of current and future climate change on species’ suitable habitats.

Predictive modeling was conducted in MaxEnt v3.4.1. Occurrence data and environmental variables for *C. punctiferalis* were processed in MaxEnt. For model calibration, 75% of occurrences were randomly selected as training data, with the remaining 25% reserved for testing. This partitioning was repeated 10 times using cross-validation. We assessed the contribution of environmental predictors to *C. punctiferalis* occurrence using Jackknife sensitivity analysis. Response curves for environment variables were generated, while for other model parameters, we retained the default settings.

Similarly to other SDMs, MaxEnt models must strike a balance between data fitting accuracy and model parsimony [17]. This equilibrium is particularly critical given their proclivity to default parameter settings [41]. To optimize MaxEnt configurations, we refined feature classes (FCs) and calibrated regularization multipliers (RMs) using the Kuenm package in R 4.4.2. Five FCs were evaluated: linear (L), quadratic (Q), product (P), threshold (T), and hinge (H), yielding 31 possible combinations. RMs were evaluated over a range from 0.5 to 4.0 at intervals of 0.5, resulting in a total of 8 RM values. Finally, 248 parameter combinations of RMs and FCs were developed, and the best MaxEnt model was chosen based on the smallest Delta AICc value. The MaxEnt model and ArcGIS v10.4.1 were used to generate a probability distribution map of *C. punctiferalis* in China. The Reclassify tool in ArcGIS was used to convert the “cloglog” format output from the MaxEnt model into binary raster data for spatial analysis. Following model evaluation using the 10th percentile training presence threshold, we categorized habitat suitability for *C. punctiferalis* into four groups: unsuitable regions (0—threshold probability of occurrence); low-suitability regions (threshold—0.40 probability of occurrence); medium-suitability regions (0.40–0.60 probability of occurrence); and high-suitability regions (>0.60 probability of occurrence). The same habitat suitability thresholds were implemented for current (1970–2000) and future (2050s and 2070s) climate models to enable comparisons.

The efficacy of a model is typically evaluated through the computation of the area under the receiver operating characteristic (ROC) curve (AUC), a metric that has been acknowledged as a sophisticated tool for appraising model validity [42]. AUC values range from 0 to 1: values approaching 1 signify stronger model performance, whereas values below 0.7 suggest that the model has lower reliability. Generally, the higher the AUC value, the more precise the model’s predictive outcomes. Within these frameworks, an AUC of 0.7–0.8 indicates moderate reliability, 0.8–0.9 indicates strong reliability, and the range of 0.9–1.0 emphasizes excellent reliability [43]. In addition, if the test AUC is closer to the training AUC, it indicates that the model results are excellent [41]. The accuracy of the model was measured using the true skill statistics (TSS = sensitivity + specificity − 1) [44]. Sensitivity refers to the probability that a species is actually present and is predicted as positive, reflecting the model’s ability to predict species distribution; specificity refers to the probability that a species is actually absent and is correctly predicted as negative, reflecting the model’s ability to predict the absence of that species.

### 2.4. Analysis of Spatial Pattern Change and Centroid Transfer

The alterations in spatial patterns are intertwined with the temporal dynamics of species’ potential suitable areas [17]. We conducted an in-depth analysis of the potential shifts in the suitable areas of *C. punctiferalis* under both current and future climate scenarios. These changes were examined by overlaying binary prediction maps across multiple time periods [45]. Specifically, taking the threshold of habitat-suitable areas as a benchmark, we employed the reclassification tools to categorize the MaxEnt results into two separate grades. Subsequently, we carried out pairwise comparisons of the outcomes under the current scenario and various environmental change scenarios. Finally, we generated a map that clearly delineates stable, expanding, and contracting areas, enabling a comprehensive assessment of the spatial distribution patterns of species’ suitable habitats over time.

We further employed spatial statistical tools in ArcGIS, using the “Mean Center” tool within the “Measuring Geographic Distribution” module to calculate the centroid of the *C. punctiferalis*’s suitable habitat across different time periods. In geographic space, the centroid is defined as the spatial average position of a suitable habitat area, i.e., the average coordinates of the pixel positions within the suitable habitat. Specifically, the centroid position is derived by computing the average spatial position of all pixels in the suitable habitat. This method enables the calculation of the spatial center of the species’ habitat under different time periods or climate scenarios. Our analysis centers on shifts in the pest’s centroid during the 2050s and 2070s across different SSP scenarios (SSP1-2.6, SSP2-4.5, SSP3-7.0, and SSP5-8.5). The migration distances of centroids in suitable areas under distinct climate scenarios were computed via SDMtoolbox-v2.4 [46].

## 3. Results

### 3.1. Model Accuracy Evaluation and Variable Selection

In this study, AUC values and TSS values were employed to assess the simulation and prediction outcomes. Model 1 achieved the best performance when the RM was set to 1 and the FC was specified as “QP”, yielding an AUC value of 0.868 ± 0.017 and a TSS value of 0.748 ± 0.013. Model 2 achieved the best performance when the RM was set to 0.5 and the FC was specified as “H”, yielding an AUC value of 0.893 ± 0.013 and a TSS value of 0.761 ± 0.009.

**Figure 3:**
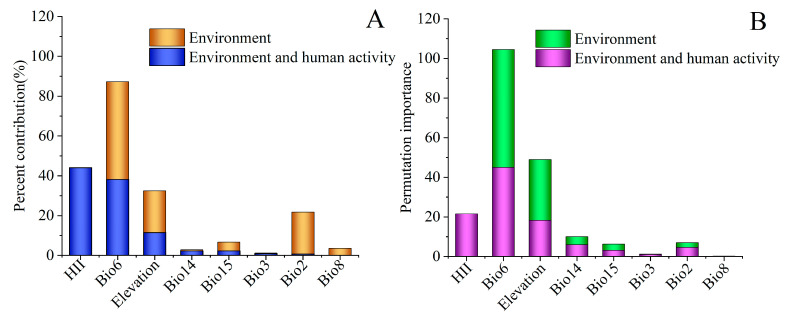
The contribution rate (**A**) and importance rate (**B**) of environmental variables in the MaxEnt model.

MaxEnt model projections showed that under the sole influence of environmental factors (Model 1), the key variables shaping the potential distribution of *C. punctiferalis* were bio6 (49.1%), bio2 (21.1%), elevation (21.0%), bio15 (4.4%), bio8 (3.6%), bio3 (0.3%), and bio14 (0.5%) (Figure 3A). Additionally, the permutation importance values for bio6, bio2, elevation, bio15, bio8, bio3, and bio14 in Model A are 59.5%, 2.3%, 30.7%, 3.1%, 0.2%, 4.0%, and 0.2%, respectively (Figure 3B). Among them, bio6, bio2, and elevation have the most pronounced effects on the species’ suitable habitats, with their cumulative contribution rate reaching 91.2%. Variable response curves illustrate the adaptive ranges of *C. punctiferalis* to these factors under the threshold, with bio6 at −17.22–22.53 °C, bio2 at 3.50–14.31 °C, and elevation at 0–1889.05 m (Figure S1).

When human interference was incorporated (Model 2), the key variables influencing *C. punctiferalis*’s potential distribution shifted to HII (44.1%), bio6 (38.2%), elevation (11.5%), bio15 (2.3%), bio14 (2.3%), bio3 (0.9%), and bio2 (0.7%) (Figure 3A), and the permutation importance values for HII, bio6, elevation, bio15, bio14, bio3, and bio2 in Model 2 were 21.6, 45.0, 18.3, 3.2, 6.0, 1.1, and 4.7, respectively (Figure 3B). Among them, HII, bio6, and elevation had the most pronounced effects on the species’ suitable habitats, with their cumulative contribution rate reaching 93.8%. Variable response curves illustrate the adaptive ranges of *C. punctiferalis* to these factors under the threshold, with HII at 12.85–68.20, bio6 at −14.78–22.09 °C, and elevation at 0–2251.13 m (Figure S2).

### 3.2. Potential Distribution of C. punctiferalis Under Current Climate and Human Interference in the Current Period

**Figure 4:**
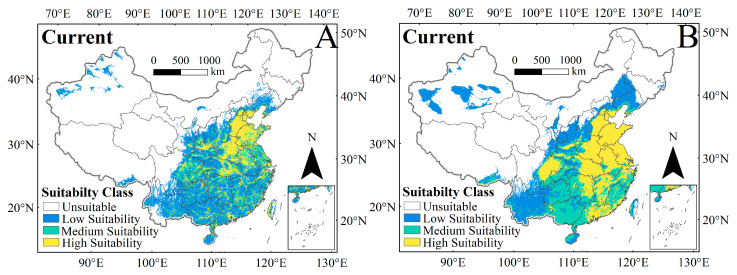
Spatial distribution of suitable habitats for *C. punctiferalis* with (**A**) and without (**B**) human activity disturbances under current climate scenarios.

The MaxEnt model was applied to predict the distribution of *C. punctiferalis* under scenarios with and without human activity disturbance (Figure 4). The suitable habitat of *C. punctiferalis* predicted by the model was consistent with its known distribution in regions such as Yunnan, Guangxi, Guangdong, Fujian, Hainan, Taiwan, Jiangxi, Hunan, Hubei, Anhui, Jiangsu, Zhejiang, Shandong, Henan, Shanxi, Shaanxi, Sichuan, Xizang, Xinjiang, Hebei, and Liaoning. These predicted habitats were classified into different suitability grades, and the area of each grade was calculated (Table 1).

Under the current climate scenario, where only environmental factors were considered, the total suitable habitat area of *C. punctiferalis* in China amounted to 3,603,591 km^2^, covering 37.54% of the country’s mainland area. The results indicated that the suitable areas were mainly distributed in multiple provinces and cities in southeastern China (Figure 4B). Specifically, the areas of high-, moderate-, and low-suitability regions were 1,222,586 km^2^, 1,014,189 km^2^, and 1,366,816 km^2^, respectively. The high-suitability region was primarily distributed across the areas of Henan, Shandong, Zhejiang, Hubei, Guangdong, Jiangxi, Anhui, Jiangsu, southern Hebei, eastern Sichuan, and eastern Hunan.

When human activity disturbances were included in the model, the total area of suitable habitats for *C. punctiferalis* in China decreased to 3,174,276 km^2^. In comparison to Model 1, the total area of suitable habitats predicted by Model 2 was reduced by 11.91%. This reduction is primarily characterized by a decrease in the areas of both moderately and highly suitable areas (Figure 4A). Specifically, the area of high suitability decreased by 39.07% to 744,908 km^2^ and the area of moderate suitability decreased by 26.72% to 743,224 km^2^.

### 3.3. Changes in the Spatial Distribution Pattern of C. punctiferalis Under Different Climate Change Scenarios

**Figure 5:**
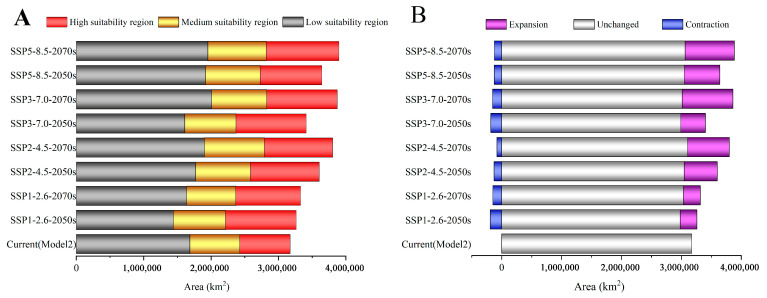
Areas of (**A**) and changes in (**B**) different suitability areas for *C. punctiferalis* during different periods in China.

We statistically analyzed the suitable habitat areas of, and changes in, *C. punctiferalis* under four climate scenarios in the 2050s and 2070s. The distribution ranges of *C. punctiferalis* under future climate conditions are basically consistent with current climate conditions, and the changes mainly reflect a significant increase in the total suitable area and the highly suitable areas (Figure 5A).

The range of suitable habitats for *C. punctiferalis* varies from 2.89% to 22.80% under four different future climate scenarios. Specifically, the average expanded area of *C. punctiferalis* is 560,591 km^2^, which represents 17.66% of the current suitable habitat areas. The decrease in suitable habitat area for this pest was not substantial, with an average reduction of 141,347 km^2^, representing just 4.45% of the current suitable habitat area. The change in suitable habitat range for *C. punctiferalis* in China shows a fluctuating increase (Figure 5B).

**Figure 6:**
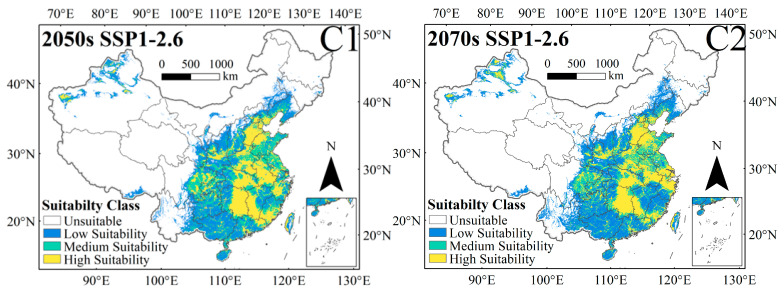

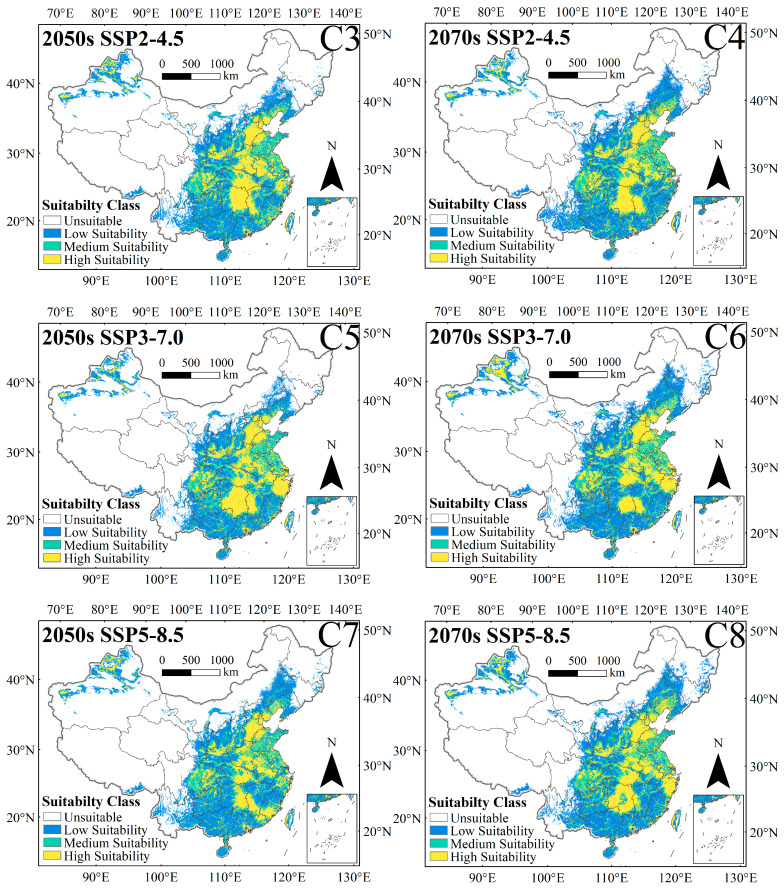
Potential habitat projections for *C. punctiferalis* under the SSP1-2.6 (**C1**,**C2**), SSP2-4.5 (**C3**,**C4**), SSP3-7.0 (**C5**,**C6**), and SSP5-8.5 (**C7**,**C8**) scenarios in the 2050s and 2070s in China.

Under the SSP1-2.6, SSP2-4.5, SSP3-7.0, and SSP5-8.5 scenarios in the 2050s, the total suitable habitat areas of *C. punctiferalis* are 3,266,075 km^2^, 3,607,075 km^2^, 3,413,899 km^2^, and 3,644,669 km^2^, respectively; compared with the current total suitable habitat area, they have increased by 2.89%, 13.63%, 7.55%, and 14.82%, respectively (Figure 6(C1,C3,C5 and C7)). Under the SSP1-2.6, SSP2-4.5, SSP3-7.0, and SSP5-8.5 scenarios in the 2070s, the total suitable habitat areas of *C. punctiferalis* are 3,330,181 km^2^, 3,805,135 km^2^, 3,872,758 km^2^, and 3,897,987 km^2^, respectively, compared with the current total suitable habitat area, they have increased by 4.91%, 19.87%, 22.00%, and 22.80%, respectively (Figure 6(C2,C4,C6 and C8)). The climate scenario with the largest growth rate for total suitable habitat area is SSP5-8.5 in the 2070s.

**Figure 7:**
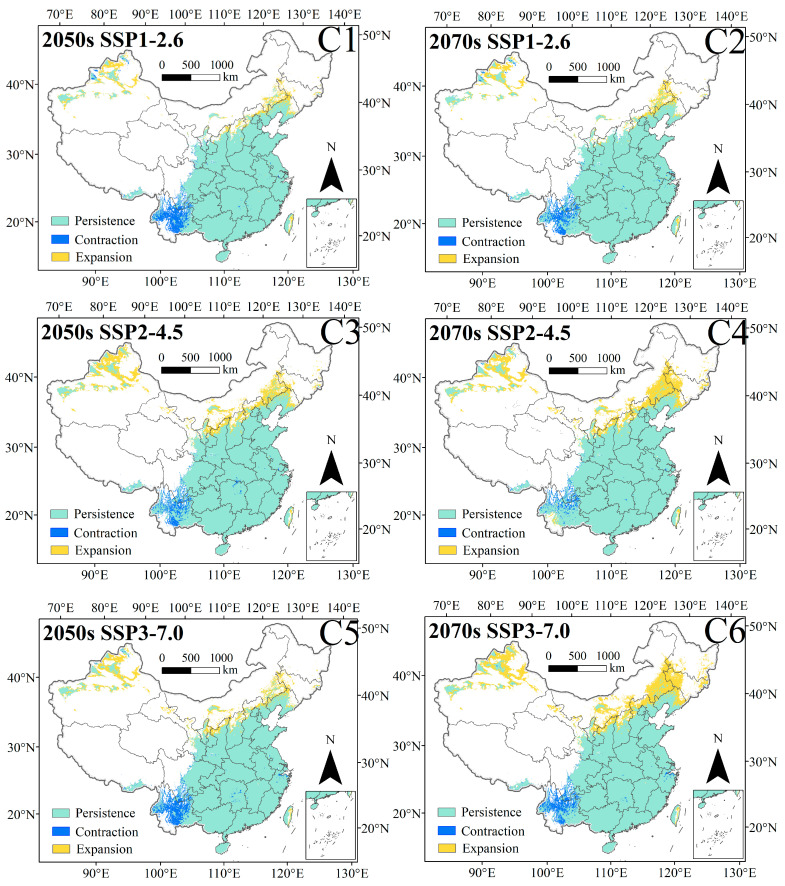

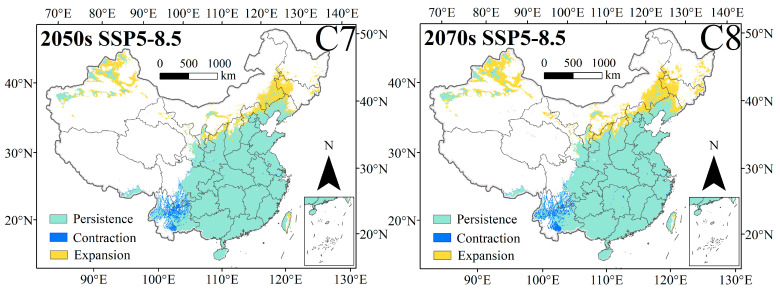
Shifts in the potential habitats for *C. punctiferalis* under the SSP1-2.6 (**C1**,**C2**), SSP2-4.5 (**C3**,**C4**), SSP3-7.0 (**C5**,**C6**), and SSP5-8.5 (**C7**,**C8**) scenarios across China during the 2050s and 2070s.

Under the SSP1-2.6, SSP2-4.6, SSP3-7.0, and SSP5-8.5 scenarios in the 2050s, the expansion areas for suitable habitat are 278,834 km^2^, 552,901 km^2^, 410,956 km^2^, and 592,085 km^2^, accounting for approximately 8.78%, 17.42%, 12.95%, and 18.65% of the current total suitable habitat area, respectively. Under different scenarios in the 2050s, the contraction areas for suitable habitat are 193,198 km^2^, 126,993 km^2^, 181,632 km^2^, and 124,532 km^2^, accounting for approximately 6.09%, 4.00%, 5.72%, and 3.92% of the current total suitable habitat area, respectively (Table 2, Figure 7(C1,C3,C5 and C7)). Under different scenarios in 2070s, the expansion areas of the suitable habitat are 282,221 km^2^, 695,362 km^2^, 848,061 km^2^, and 824,306 km^2^, respectively, accounting for approximately 8.89%, 21.91%, 26.72%, and 25.97% of the current total suitable habitat area, respectively. Under different scenarios in the 2070s, the contraction areas for suitable habitat are 148,595 km^2^, 80,491 km^2^, 153,237 km^2^, and 122,095 km^2^, accounting for approximately 4.68%, 2.54%, 4.83%, and 3.85% of the current total suitable habitat area, respectively (Table 2, Figure 7(C2,C4,C6 and C8)).

### 3.4. Transfer of the Potential Distribution of C. punctiferalis

**Figure 8:**
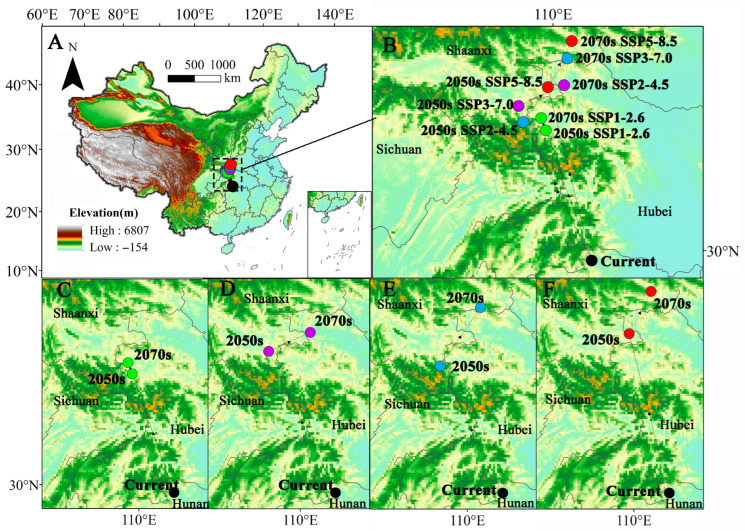
(**A**,**B**) Potential distribution center of *C. punctiferalis* under SSP1-2.6 (**C**), SSP2-4.5 (**D**), SSP3-7.0 (**E**), and SSP5-8.5 (**F**).

Under the current climate conditions, the distribution center of *C. punctiferalis* is situated in Shimen County, Changde City, Hunan Province, with the coordinates 29.8663 N, 110.7667 E. Under the four SSPs, in the 2050s and 2070s, the centroids of future suitable habitats will generally show a northward shifting trend compared with their current positions. We also found that the future distribution centroids of *C. punctiferalis* will shift within the range of 24.91 to 336.87 km. Overall, from the present day to the 2050s, the distance of distribution centroid migration is relatively long, while from the 2050s to the 2070s, the distance of centroid migration is relatively short (Figure 8A,B).

In the SSP1-2.6 scenario, the center of the distribution area of *C. punctiferalis* will experience notable shifts (Figure 8C). In the 2050s, it will shift 260.21 km in the northwest direction and reach Zhuxi County, Shiyan City, Hubei Province, with the new coordinates being 32.0713 N, 109.8527 E. By the 2070s, it will then shift a substantial 24.91 km in the northwest direction and arrive at Zhuxi County, Shiyan City, Hubei Province, at the coordinates 32.2803 N, 109.7574 E.

In the SSP2-4.5 scenario, the center of the distribution range of *C. punctiferalis* is set to undergo significant displacements over time (Figure 8D). In the 2050s, it will relocate 323.05 km in the northwest direction, arriving at Pingli County, Ankang City, Shaanxi Province, with the coordinates 32.4829 N, 109.2908 E. In the 2070s, it will shift 95.72 km in the northeast direction and reach Yunxi County, Shiyan City, Hubei Province, with the new coordinates being 32.8347 N, 110.2240 E.

In the SSP3-7.0 scenario, the displacement trend of the center of the distribution range of *C. punctiferalis* over time is similar to that in the SSP2-4.5 scenario (Figure 8E). In the 2050s, it will relocate 291.74 km in the northwest direction, arriving in Pingli County, Ankang City, Shaanxi Province, with the coordinates 32.2107 N, 109.3918 E. As time progresses to the 2070s, the center will then move 146.67 km in the northeast direction and arrive in Shanyang County, Shangluo City, Shaanxi Province, with the new location being 33.2874 N, 110.2978 E.

In the SSP5-8.5 scenario, the center of the distribution area of *C. punctiferalis* will undergo migration in different directions, just like in the previous two scenarios (Figure 8F). In the 2050s, the center will shift an even greater distance, specifically 336.87 km in the northwest direction, reaching Baihe County, Ankang City, Shaanxi Province, at 32.8026 N, 109.8934 E. By the 2070s, it will move 97.85 km in the northeast direction and arrive in Danfeng County, Shangluo City, Shaanxi Province, with the new coordinates being 33.5796 N, 110.3872 E.

## 4. Discussion

The optimal MaxEnt model reveals that under climate change and human interference, the distribution range of *C. punctiferalis* is most affected by three factors: HII, bio6, and elevation. Human activities are a key factor affecting the dynamic distribution of species. Their impact on species distribution is mainly manifested through promoting [47] and inhibiting effects [48,49]. The current study reveals that the area of highly suitable habitats for *C. punctiferalis* has narrowed by 39.07% under human activity disturbance, inferring that human activities have impeded the expansion of this pest. This may be attributed to the following factors: Firstly, intensive agricultural management measures, especially the promotion of mulching cultivation for field crops [50,51,52], fruit-bagging technology [53,54], and strict winter field cleaning and crop rotation systems [55,56], have significantly reduced pests’ population size and chance of survival. To be specific, the techniques of bagging and mulching can prevent and control the occurrence of *C. punctiferalis* by targeting critical stages in its life cycle. Fruit bagging exerts a direct preventive and control effect on *C. punctiferalis*. Adult moths of this species typically select intact, fresh parts of fruit surfaces for oviposition (egg-laying). Fruit bags can create a physical barrier around the fruit, directly blocking the contact path between adult moths and the fruit. This prevents adult female moths from accessing the fruit and laying eggs, thereby reducing the pest population [57]. Mulching mainly inhibits the occurrence of *C. punctiferalis* indirectly through microenvironment regulation and chemical interference. By covering the ground with plastic film, the temperature and humidity conditions of the soil surface can be modified [58]. For example, in summer, this technique reduces the surface temperature, reduces water evaporation, disrupts the hatching environment for eggs of harmful insects in the topsoil, and lowers the hatching rate of larvae. Studies have shown that environmental factors such as temperature and humidity have a significant impact on growth, development, reproduction, and egg hatching in *C. punctiferalis*. Additionally, certain chemical components (e.g., terpenoids and phenols) contained in some mulching film materials, as well as the volatile gases released during their decomposition, can interfere with the pest’s chemical communication system [59]. This impairs the pests’ ability to locate host plants, thereby reducing the risk of crop damage. Additionally, film mulching can inhibit the growth of weeds in orchards [60]. Since weeds serve as one of the intermediate hosts of *C. punctiferalis*, reducing weed growth can indirectly lower the pest’s population base and assist in enhancing the pest control effect. Secondly, the availability of pest habitats has decreased due to land use changes [61]. Human activities are often concentrated in the native habitats of species, resulting in the fragmentation and degradation of habitats, thereby restricting the suitable distribution areas of species [26]. Additionally, human activities may further constrain species’ ranges via adverse effects, such as habitat destruction and pollution [62]. The widespread use of high-efficiency pesticides, along with the application of green pest control methods such as biological control and sex pheromone technology, has effectively controlled pest populations while reducing reliance on pesticides [32]. Despite the potential impact of climate change, the current active prevention and control measures implemented by humans represent the dominant force in curbing the expansion of pest distribution, even leading to a reduction in some confined areas.

However, it cannot be ignored that human activity-mediated transportation, migration, and commercial activities have promoted the introduction of species to new remote regions and accelerated their spatial expansion [63,64]. Moreover, the current “reduction” is usually relative to a lack of effective prevention and control measures or refers to certain areas where severe infestations once occurred but which are now under effective control.

Environmental variables such as temperature, precipitation, and elevation exert either direct or indirect effects on the survival of insect species [65]. Numerous studies have found that temperature significantly affects the distribution, dispersal, and population density of insects [66,67]. Prior research has also indicated that the minimum temperature in the coldest month influences the potential geographical distributions of insects [68,69]. Our findings also revealed that this variable emerged as the key factor affecting the survival and development of *C. punctiferalis*. Research has found that temperature significantly influences the performance of *C. punctiferalis* across the entire life cycle [30]. In particular, lower temperatures may not directly inhibit the population but they can prolong the developmental period of larvae [70]. It is worth noting that *C. punctiferalis* is an insect that overwinters as diapause larvae, and low temperature plays a significant role in the initiation of diapause. A recent report has shown that all *C. punctiferalis* larvae enter diapause, regardless of exposure to long-day or short-day photoperiods at 20 °C; in contrast, the diapause rate is consistently below 3% at 30 °C [71]. The interplay between temperature and other factors (such as elevation and precipitation) may also exert an indirect influence on the survival and development of insects [72,73]. Consistent with our findings, previous research has also revealed that elevation affects the potential geographical distribution of insects [74,75]. Elevation plays a significant role in the adaptive evolution and distribution of species, typically by influencing water and energy availability and/or functioning as a dispersal barrier [66,76,77]. In addition, elevation is negatively correlated with temperature, which can further affect the distribution of pests by affecting the redistribution of water and heat [78].

Under current conditions, the model-predicted suitable habitats for *C. punctiferalis* aligned with its known distribution areas, mainly concentrated in southern Northeast China, North China, the Yangtze River Basin, and southern regions beyond it. The predicted results showed that the highly suitable habitats for this pest are basically consistent with the main maize-producing areas in the Huang-Huai-Hai region of China. After incorporating the HII variable, the results indicate that the area of highly suitable habitats has decreased; however, compared with the prediction results without the HII variable, the overall distribution pattern in China has not changed. These results suggest that under current conditions, there is a stable risk of *C. punctiferalis* outbreaks, especially in the maize-growing areas of the Huang-Huai-Hai region in China.

Compared with the current period (Model 2), the area of suitable habitats under future climate scenarios is expected to increase by approximately 91,799 km^2^ to 723,711 km^2^ and the centroids of future suitable habitats will generally show a northward shifting trend compared with their current positions. These results indicate that climate change will continue to offer highly favorable conditions for *C. punctiferalis* within its originally suitable areas. These findings align with prior research projections, which have already suggested that insect ranges may shift toward higher latitudes as a response to the challenges brought about by global warming and climate change [79,80]. This could be due to significant climate shifts amid global warming, resulting in insects displaying notable physiological plasticity, which enables them to achieve higher reproductive rates and stronger dispersal capacities [81,82]. The northward shift in the distribution centroid of *C. punctiferalis* will trigger a series of ecological and agricultural reactions that are both complex and significant. In terms of invasion risks, the original climatic conditions in northern regions previously restricted the survival and reproduction of *C. punctiferalis*, maintaining relatively stable local ecosystems. However, with its northward migration, the probability of *C. punctiferalis* establishing populations in new northern areas has increased significantly. On one hand, the species and quantity of natural enemies in these newly invaded regions differ from those in the moth’s original suitable habitats, potentially failing to control the rapidly expanding *C. punctiferalis* population. On the other hand, northern ecosystems have relatively simple structures and low stability, lacking the self-regulating capacity to buffer the impact of newly invasive species. This means that once *C. punctiferalis* establishes itself, it may spread rapidly, disrupting the local ecological balance. In terms of interactions with host plants, the northward centroid shift has led to a notable change in the host range of *C. punctiferalis*. Beyond some overlapping host species, cold-tolerant crop varieties unique to northern regions (e.g., certain cold-resistant fruit trees) have become new potential hosts. *C. punctiferalis* may adapt to these new hosts by adjusting its behaviors (such as changing egg-laying locations and modifying larval feeding preferences) and evolving physiological traits (such as developing mouthpart structures adapted to new hosts and optimizing its digestive enzyme systems). This adaptation process will not only promote the expansion of the *C. punctiferalis* population itself but also directly reduce the yield and quality of host crops in northern regions, threatening the growth security of these crops. In terms of impacts on agricultural production, the northward centroid shift for *C. punctiferalis* poses severe challenges to northern agriculture. Northern China is a key production area for grains and fruits, with the extensive cultivation of crops such as corn, apples, and pears. Infestations by *C. punctiferalis* will directly cause crop yield reductions, affecting farmers’ incomes and the stability of the agricultural product industrial chain. To prevent and control this pest, more human and material resources must be invested in agricultural production, which will not only increase planting costs but may also exacerbate ecological damage in northern regions due to the excessive use of pesticides.

As a polyphagous and boring pest, *C. punctiferalis* has a wide range of hosts that provide it with abundant food and living space, further increasing the difficulty of its control. Our study reveals the spatial distribution patterns and dynamic change trends of *C. punctiferalis*, laying an important foundation for its scientific, precise, and sustainable control. Firstly, accurate distribution prediction provides a scientific basis for formulating regionalized control strategies. After clarifying potential suitable habitats and diffusion paths through model prediction, key control areas can be targeted: in highly suitable areas, intensive monitoring and early interventions should be implemented, such as emergency measures combining sex pheromone trapping with chemical control; in moderately and lowly suitable areas, ecological regulation should be the main approach, including adjusting crop-planting structures and protecting natural enemies to build ecological barriers, thereby reducing the excessive use of chemical pesticides. To be specific, in traditional high-pest-incidence areas in Central China (e.g., the major peach and corn producing regions in Hunan and Hubei provinces), integrated prevention and control measures such as “paper bagging + straw mulching” should be implemented 10–15 days after peach trees finish blooming. Before bagging, *Bacillus thuringiensis* (Bt) should be sprayed to reduce the initial egg quantity; in corn fields, intercropping with leguminous plants could be adopted to enhance the pest control capacity of natural enemies. For the potential pest expansion areas in North China (e.g., the new fruit-growing regions in Henan and Shandong provinces), orchard soil should be plowed in early March to destroy overwintering pupae. Additionally, the large-scale continuous planting of host plants such as corn around orchards should be avoided, and non-host plant isolation belts should be established to slow down pest spread. In the complex mountainous areas of Southwest China (e.g., the sloping orchards in Sichuan and Guizhou provinces), sex pheromone traps can be set up in low-altitude intercropping areas to monitor adult moths and enable precise pesticide application. For scattered orchards in high-altitude areas, priority should be given to planting insect-resistant varieties to reduce the impact of chemical control on the mountainous ecosystem. This differentiated strategy could not only improve control efficiency but also reduce damage to agricultural ecosystems. Secondly, dynamic distribution prediction helps optimize the temporal and spatial allocation of control resources. In traditional control, an insufficient grasp of pest occurrence dynamics often leads to delayed control timing or resource wastage. However, the distribution prediction model based on climatic factors can predict in advance the occurrence peaks and diffusion directions of *C. punctiferalis*, allowing concentrated control during key time nodes (e.g., the adult eclosion period or larval hatching period). Meanwhile, monitoring equipment, human resources, and pesticide resources can be rationally allocated according to the predicted spatial distribution characteristics. For example, setting up monitoring points along the predicted migration routes can achieve the early interception of pest sources, significantly reducing the risk of outbreaks. In addition, long-term research on distribution prediction provides forward-looking support for pest management in the context of climate change. This study simulates distribution dynamics under different climate change scenarios, which can provide a basis for formulating adaptive control strategies, for example, adjusting crop-planting layouts in advance to avoid newly emerging suitable areas or breeding insect-resistant varieties to cope with the potential trend of intensified damage.

It is worth noting that we restricted our analysis to the impacts of bioclimatic variables and human activity on species distribution; however, species distribution is also shaped by a range of biological factors (e.g., interspecific competition, predation, and disease) and abiotic factors (e.g., soil, topography, and solar radiation) [83]. Future studies should prioritize the integration of biological data, including host plant distribution and natural enemy population density. On one hand, data supplementation through public database retrieval and regional joint surveys can enhance the accumulation of biological datasets. On the other hand, field validation (e.g., analyzing the correlation between biotic factors and *C. punctiferalis* population size) can be used to calibrate model parameters. Based on these efforts, model prediction analyses can be conducted to further improve the ecological relevance and reliability of prediction results. In addition, future climate change is accompanied by considerable uncertainties, so the exclusive use of the BCC-CMS2-MR GCM to simulate potential suitable habitats for *C. punctiferalis* may enhance the instability of MaxEnt model predictions. This could lead to significant inaccuracies in the projected geographic range and habitat occurrence for *C. punctiferalis*, thus hindering decision-makers from developing effective ecological protection strategies. Explaining the impact of all these factors requires a more comprehensive niche modeling approach, as demonstrated by previous researchers [84,85] who employed multiple GCMs for simulation and prediction, significantly reducing prediction uncertainty and errors. The development of models for predicting potential suitable habitats for *C. punctiferalis* in the future should refer to these key insights.

## 5. Conclusions

In this research, we employed an optimized MaxEnt model to model the current and future distribution of suitable habitats for *C. punctiferalis* under climate change. Under current climatic conditions, 37.54% of China’s area represents a suitable habitat for *C. punctiferalis*. When human activity disturbances were included in the model, the total area of suitable habitats for *C. punctiferalis* accounted for 33.07% of China’s total area. Therefore, human activities play a crucial role in predicting the distribution of this pest and have an inhibitive effect on its spread, which cannot be ignored when predicting future distribution. Under future climate conditions, suitable habitats for *C. punctiferalis* are expected to increase to varying degrees, with a potential increase of 22.80% by the 2070s, especially under the worst-case scenario (SSP5-8.5). Additionally, the pest’s potential distribution range is projected to move toward high-latitude areas, and the distribution center is expected to exhibit a slight northward shift. In summary, our study reveals the impacts of human activities and climate change on the distribution patterns of *C. punctiferalis* and can provide a reference for the monitoring, early warning, and prevention of *C. punctiferalis*.

## Figures and Tables

**Table 1:** The area of suitable habitat of *C. punctiferalis* with and without human activity disturbance (km^2^).

Human Activity	Highly Suitable Habitat	Moderately Suitable Habitat	Low Suitable Habitat	Unsuitable Habitat
Without human activity	1,222,586	1,014,189	1,366,816	5,996,409
With human activity	744,908	743,224	1,686,145	6,425,724

**Table 2:** Rates of change in the suitable areas of *C. punctiferalis* during different periods under four climate scenarios.

Climate Scenario	Decades	Predicted Area (km^2^) and % of the Corresponding Current Area						
		Total Suitable Region	Contraction	Unchanged	Expansion	Range Change	Contraction Percentage	Expansion Percentage
	1970–2000	3,174,276	–	–	–	–	–	–
SSP1-2.6	2050s	3,266,075	193,198	2,980,340	278,834	2.89%	6.09%	8.78%
	2070s	3,330,181	148,595	3,035,986	282,221	4.91%	4.68%	8.89%
SSP2-4.5	2050s	3,607,075	126,993	3,046,769	552,901	13.63%	4.00%	17.42%
	2070s	3,805,135	80,491	3,103,696	695,362	19.87%	2.54%	21.91%
SSP3-7.0	2050s	3,413,899	181,632	2,991,672	410,956	7.55%	5.72%	12.95%
	2070s	3,872,758	153,237	3,013,798	848,061	22.00%	4.83%	26.72%
SSP5-8.5	2050s	3,644,669	124,532	3,049,247	592,085	14.82%	3.92%	18.65%
	2070s	3,897,987	122,095	3,062,109	824,306	22.80%	3.85%	25.97%

## Data Availability

The authors confirm that all data are available in this paper.

## Supplementary Materials

The following supporting information can be downloaded at: https://www.mdpi.com/article/10.3390/insects16100998/s1, Table S1: Pearson correlation analysis between environmental factors (Model 2). Figure S1: Response curves of *C. punctiferalis* to the environmental variables with the highest contribution to model building (Model 1). Figure S2: Response curves of *C. punctiferalis* to the environmental variables with the highest contribution to model building (Model 2).
