# Supplementary material for: Impacts of Climate Change and Human Activity on the Potential Distribution of Conogethes punctiferalis in China
Source: Insects. 2025 Sep 25;16(10):998. doi: 10.3390/insects16100998 (PMC12565121; doi:10.3390/insects16100998)

Table S1. Pearson correlation analysis between environmental factors (Model2).

Pearson correlation	Bio1	Bio2	Bio3	Bio4	Bio5	Bio6	Bio7	Bio8	Bio9	Bio10	Bio11	Bio12	Bio13	Bio14	Bio15	Bio16	Bio17	Bio18	Bio19	Elevation	HII
Bio1	1	-0.649	0.294	-0.742	0.447	0.931	-0.776	0.530	0.953	0.645	0.943	0.779	0.755	0.555	-0.387	0.768	0.589	0.703	0.613	-0.270	-0.053
Bio2	-0.649	1	0.200	0.631	-0.041	-0.784	0.764	-0.184	-0.684	-0.286	-0.695	-0.747	-0.675	-0.654	0.519	-0.681	-0.624	-0.657	-0.607	0.117	0.120
Bio3	0.294	0.200	1	-0.606	-0.343	0.350	-0.460	-0.180	0.371	-0.301	0.456	0.246	0.282	-0.109	0.055	0.333	-0.080	0.309	-0.044	0.540	-0.201
Bio4	-0.742	0.631	-0.606	1	0.23	-0.911	0.980	0.041	-0.834	0.026	-0.922	-0.795	-0.738	-0.471	0.414	-0.786	-0.468	-0.729	-0.475	-0.365	0.265
Bio5	0.447	-0.041	-0.343	0.230	1	0.141	0.190	0.707	0.270	0.936	0.145	0.061	0.026	0.238	-0.149	0.001	0.281	-0.063	0.292	-0.762	0.246
Bio6	0.931	-0.784	0.350	-0.911	0.141	1	-0.945	0.276	0.952	0.365	0.990	0.861	0.804	0.609	-0.479	0.832	0.615	0.768	0.624	0.001	-0.158
Bio7	-0.776	0.764	-0.460	0.980	0.190	-0.945	1	-0.040	-0.855	-0.053	-0.934	-0.834	-0.788	-0.525	0.426	-0.825	-0.518	-0.783	-0.522	-0.253	0.239
Bio8	0.530	-0.184	-0.180	0.041	0.707	0.276	-0.040	1	0.314	0.838	0.287	0.076	0.228	-0.020	0.242	0.175	0.023	0.275	0.052	-0.790	0.275
Bio9	0.953	-0.684	0.371	-0.834	0.270	0.952	-0.855	0.314	1	0.470	0.962	0.852	0.790	0.65	-0.501	0.818	0.684	0.731	0.713	-0.085	-0.132
Bio10	0.645	-0.286	-0.301	0.0262	0.936	0.365	-0.053	0.838	0.470	1	0.362	0.262	0.281	0.317	-0.127	0.245	0.370	0.210	0.392	-0.834	0.226
Bio11	0.943	-0.695	0.456	-0.922	0.145	0.990	-0.934	0.287	0.962	0.362	1	0.844	0.800	0.561	-0.430	0.831	0.579	0.765	0.596	0.0167 78835	-0.159
Bio12	0.779	-0.747	0.246	-0.795	0.061	0.861	-0.834	0.076	0.852	0.262	0.844	1	0.922	0.789	-0.556	0.947	0.802	0.847	0.810	0.020	-0.253
Bio13	0.755	-0.675	0.282	-0.738	0.026	0.804	-0.788	0.228	0.790	0.281	0.800	0.922	1	0.555	-0.225	0.992	0.585	0.946	0.615	-0.102	-0.196
Bio14	0.555	-0.654	-0.109	-0.471	0.238	0.609	-0.525	-0.020	0.650	0.317	0.561	0.789	0.555	1	-0.786	0.587	0.978	0.458	0.956	-0.072	-0.173
Bio15	-0.387	0.519	0.0546	0.414	-0.149	-0.479	0.426	0.242	-0.501	-0.127	-0.430	-0.556	-0.225	-0.786	1	-0.293	-0.763	-0.153	-0.724	-0.210	0.210
Bio16	0.768	-0.681	0.333	-0.786	0.001	0.832	-0.825	0.175	0.818	0.245	0.831	0.947	0.992	0.587	-0.293	1	0.609	0.946	0.637	-0.027	-0.226
Bio17	0.589	-0.624	-0.080	-0.468	0.281	0.615	-0.518	0.023	0.684	0.370	0.579	0.802	0.585	0.978	-0.763	0.609	1	0.457	0.989	-0.124	-0.164
Bio18	0.703	-0.657	0.309	-0.729	-0.063	0.768	-0.783	0.275	0.731	0.210	0.765	0.847	0.946	0.458	-0.153	0.946	0.457	1	0.481	-0.059	-0.184
Bio19	0.613	-0.607	-0.044	-0.475	0.292	0.624	-0.522	0.052	0.713	0.392	0.596	0.810	0.615	0.956	-0.724	0.637	0.989	0.481	1	-0.148	-0.144
Elevation	-0.270	0.117	0.540	-0.365	-0.762	0.001	-0.253	-0.790	-0.085	-0.834	0.017	0.020	-0.102	-0.072	-0.210	-0.027	-0.124	-0.059	-0.148	1	-0.327
HII	0.053	0.120	-0.201	0.265	0.246	-0.158	0.239	0.275	-0.132	0.226	-0.159	-0.253	-0.196	-0.173	0.210	-0.226	-0.164	-0.184	-0.144	-0.327	1

Figure. S1. Response curves of *C. punctiferalis* to the environmental variables with the highest contribution to model building (Model1).

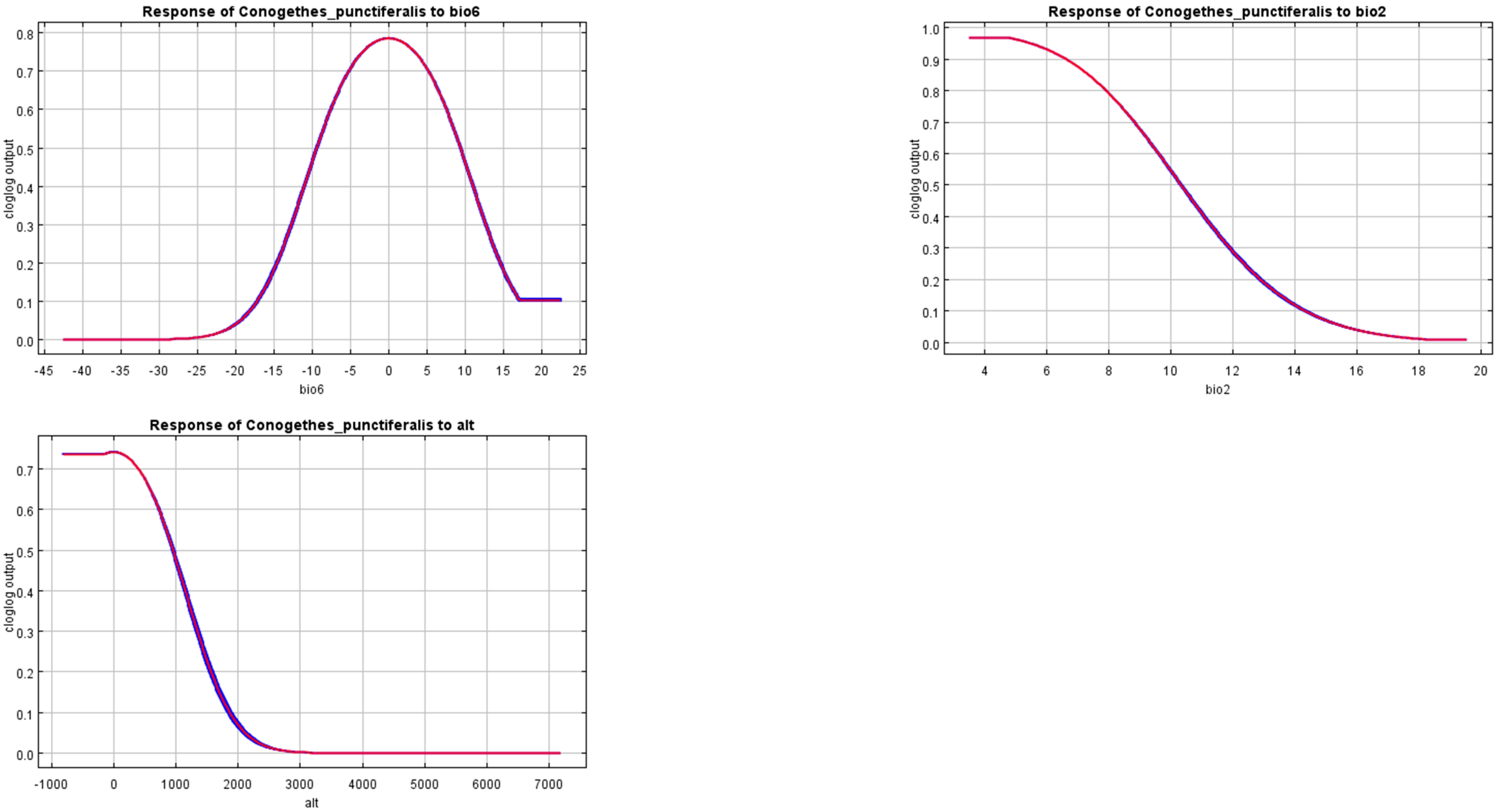

Figure. S2. Response curves of *C. punctiferalis* to the environmental variables with the highest contribution to model building (Model2).

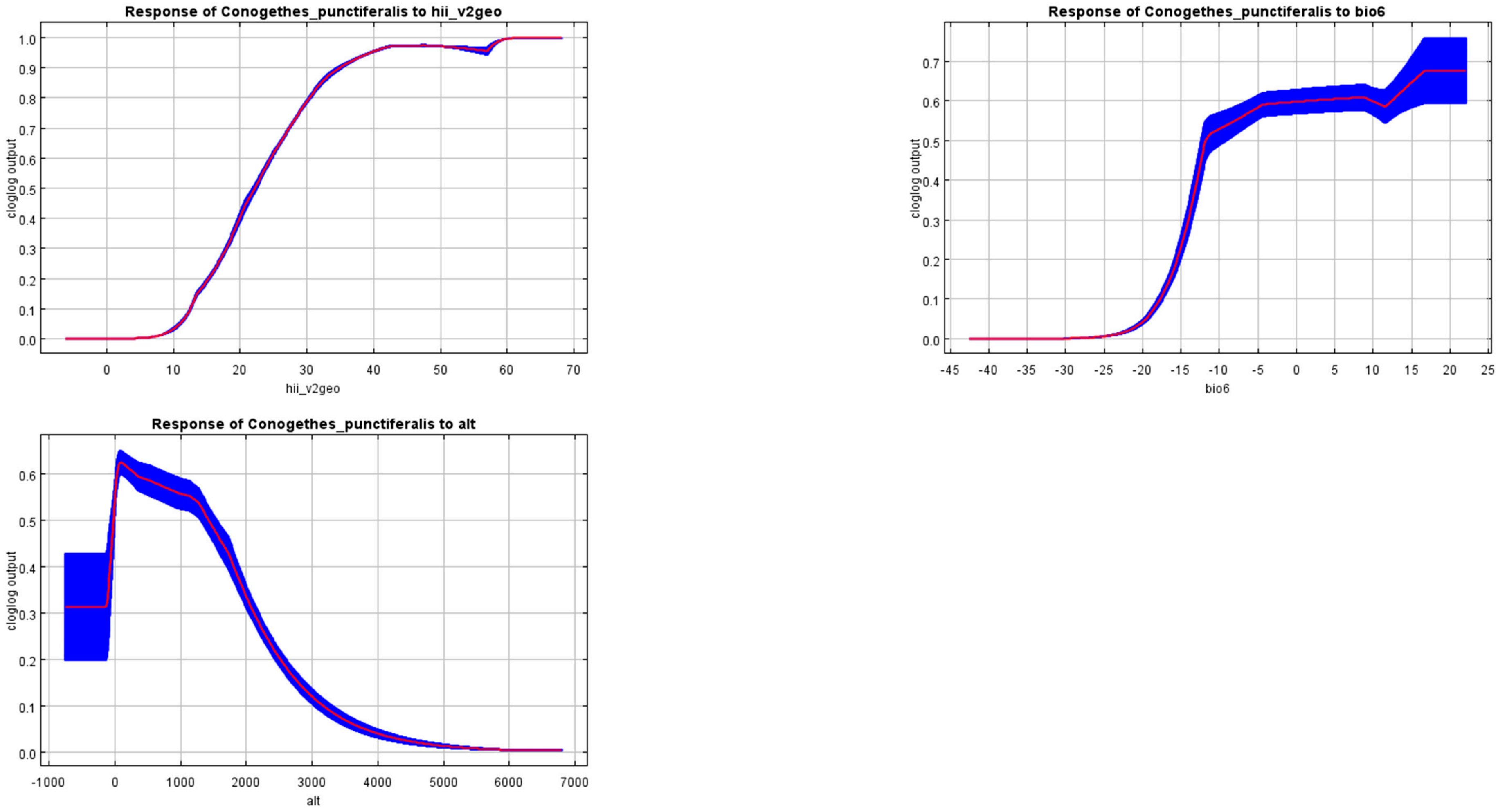

Supplement: Supplementary file 1 [file insects-16-00998-s001.zip › insects-3845314-supplementary.pdf]
